# On-Tissue Chemical Derivatization for Mass Spectrometry Imaging of Fatty Acids with Enhanced Detection Sensitivity

**DOI:** 10.3390/biom15030366

**Published:** 2025-03-03

**Authors:** Malik Ebbini, Zicong Wang, Hua Zhang, Kelly H. Lu, Penghsuan Huang, Cameron J. Kaminsky, Luigi Puglielli, Lingjun Li

**Affiliations:** 1 School of Pharmacy, University of Wisconsin-Madison, Madison, WI 53705, USA; ebbini@wisc.edu (M.E.); zwang2553@wisc.edu (Z.W.); 2 Department of Chemistry, University of Wisconsin-Madison, Madison, WI 53706, USA; khlu2@wisc.edu (K.H.L.); penghsuan.huang@wisc.edu (P.H.);; 3 Department of Medicine, University of Wisconsin-Madison, Madison, WI 53705, USA; lp1@medicine.wisc.edu; 4 Waisman Center, University of Wisconsin-Madison, Madison, WI 53705, USA; 5 Geriatric Research Education Clinical Center, Veterans Affairs Medical Center, Madison, WI 53705, USA; 6 Lachman Institute for Pharmaceutical Development, School of Pharmacy, University of Wisconsin-Madison, Madison, WI 53705, USA; 7 Wisconsin Center for NanoBioSystems, School of Pharmacy, University of Wisconsin-Madison, Madison, WI 53705, USA

**Keywords:** on-tissue chemical derivatization, MALDI, mass spectrometry imaging, fatty acids, Alzheimer’s disease mouse model

## Abstract

The dysregulation of fatty acid (FA) metabolism is linked to various brain diseases, including Alzheimer’s disease (AD). Mass spectrometry imaging (MSI) allows for the visualization of FA distribution in brain tissues but is often limited by low detection sensitivity and high background interference. In this work, we introduce a novel on-tissue chemical derivatization method for FAs using Girard’s Reagent T (GT) as a derivatization reagent combined with 2-chloro-1-methylpyridinium iodide (CMPI) as a coupling reagent and triethylamine (TEA) to provide a basic environment for the reaction. This method significantly enhances the detection sensitivity of FAs, achieving a 1000-fold improvement over traditional negative ion mode analysis. Our method enabled us to observe a notable depletion of oleic acid in the corpus callosum of AD mouse model brain tissue sections compared to wild-type control brain tissue sections. The reliability of our method was validated using LC-MS/MS, which confirmed the presence of eight distinct GT-labeled FAs across various tissue locations. This approach not only improves FA detection in brain tissues but also has the potential to provide a deeper understanding of FA dynamics associated with AD pathogenesis.

## 1. Introduction

Fatty acids (FAs) are fundamental to brain function and play a significant role in the pathology of brain-related diseases [1,2]. They are crucial in energy metabolism [3] and maintaining the structural integrity of cell membranes. The dynamics of how FAs are integrated into or released from phospholipids significantly influence membrane fluidity (as FAs with a high unsaturation number lead to increased membrane fluidity), which is known to be altered in disease [4]. Furthermore, FAs serve pivotal roles in cellular signaling pathways, both as direct signaling molecules [5] and as precursors to other bioactive compounds [6]. Consequently, the abundance of FA molecules within the brain affects cell signaling, membrane fluidity, and energy metabolism.

Note that both the abundance and spatial distribution of FAs are critical factors. Given the brain’s complexity and heterogeneity, understanding the spatial distribution of FAs across different brain regions is essential for comprehending the progression of Alzheimer’s disease (AD). Traditional studies employing cerebral glucose metabolism and brain atrophy markers have underscored pronounced regional variations in AD progression [7,8,9,10]. Hu et al. compared different brain regions and found that the olfactory bulb has the highest amount of FAs, while a low amount of FAs is found in the cerebellum [11]. It is also important to note that different FA species localize in different regions. For example, docosahexaenoic acid localizes in gray matter [12], and oleic acid localizes in white matter [13]. Hence, techniques that offer spatial resolution are indispensable for capturing this spatial diversity. Matrix-assisted laser desorption/ionization mass spectrometry imaging (MALDI-MSI) provides a robust platform for this purpose. It facilitates the visualization of biomolecule distributions within tissue sections without the need for homogenization, thereby preserving the intrinsic heterogeneity of the tissues [14]. This makes MALDI-MSI ideally suited for the detailed localization of various biomolecules [15,16,17,18,19,20].

Although MALDI-MSI can provide spatial localization information, there are limitations in its detection sensitivity when working with complex samples. Indeed, it is well established that the higher the spatial resolution, the lower the detection sensitivity [21]. On-tissue chemical derivatization is emerging as a promising strategy for enhancing the detection sensitivity in MSI [22,23,24,25]. For instance, a range of derivatization reagents have been applied to label FAs prior to MSI; derivatization reagents that have been used for FAs include 2-picolylamine [22], *N*,*N*-dimethylpiperazine iodide [23], *N*,*N*,*N*-trimethyl-2-(piperazin-1-yl)ethan-1-aminium iodide [24], and (1-(4-(aminomethyl)phenyl)pyridin-1-ium chloride [25]. However, none of these derivatization methods have all three of the following advantages: (1) a demonstrated ability to perform imaging of both FAs and phospholipids simultaneously, (2) a derivatization reagent with a permanent positive charge, and (3) commercially available reagents that do not require custom synthesis. Each of the previous methods meets two out of three criteria, but none of them meets all the stated criteria. We sought to develop a derivatization method that meets all three criteria.

In this study, we introduce a novel on-tissue chemical derivatization method using Girard’s Reagent T (GT) to label long-chain FAs in situ on AD mouse brain tissue sections. GT has been used for on-tissue labeling of aldehydes and ketones [26,27,28]. Note that GT has been used for in-solution labeling of short-chain FAs [29]. However, to our knowledge, GT has not been used for on-tissue labeling of long-chain FAs before. Our research demonstrates that GT labeling can add permanent positive charges to FAs, significantly enhancing the detection sensitivity in MALDI-MSI. This method was applied to compare FA profiles in wild-type (WT) mouse brain tissue sections and APP/PS1 AD transgenic mouse model brain tissue sections, showcasing the potential of our method to reveal AD-related metabolic alterations.

## 2. Materials and Methods

### 2.1. Materials and Reagents

Optima-grade methanol, optima-grade water, optima-grade acetonitrile (ACN), optima-grade 2-propanol (IPA), and formic acid were purchased from Fisher Scientific (Pittsburgh, PA, USA). FA standards—which include FA 16:1, FA 18:1, and FA 20:1—were purchased from Sigma Aldrich (St. Louis, MO, USA). Indium tin oxide (ITO, part number: CB-90IN-S107, 25mm × 75 mm × 1 mm)-coated glass slides were purchased from Delta Technologies, LTD (Loveland, CO, USA). 2,5-dihyxdroxybenzoic acid (DHB), α-cyano-4-hydroxycinammic acid (CHCA), 9-aminocridine (9-AA), Girard Reagent T (GT), trifluoroacetic acid (TFA), chloroform, lyso phosphatidylglycerol 20:1 (lyso PG 20:1) standard, and phosphatidylcholine 36:2 (PC 36:2) standard were purchased from Sigma Aldrich (St. Louis, MO, USA). Triethylamine (TEA) was purchased from Alfa Aesar (Haverhill, MA, USA). 2-chloro-1-methylpyridinium iodide (CMPI) was purchased from Chem-Impex Int’l Inc. (Wood Dale, IL, USA), https://www.sigmaaldrich.com/US/en/product/sial/70990 (accessed on 26 December 2024).

All animals used in this study are *Mus musculus* strain C57BL/6 J. APP695/swe/PS1-dE9 (APP/PS1) double-transgenic mice were obtained from Jackson Laboratory (MMRRC Stock No. 34832-JAX). The mice were housed in standard cages provided by the University Laboratory Animal Resources and grouped with littermates, with 1–5 mice per cage. The animals were supplied standard chow and water ad libitum. Animal experiments were performed in accordance with the National Institutes of Health Guide for the Care and Use of Laboratory Animals and were approved by the Institutional Animal Care and Use Committee of the University of Wisconsin-Madison (protocol #M005120). 

A 10-month-old healthy male mouse brain was embedded with 10% gelatin in water. Only the bottom half of the brain was embedded; the top of the brain was visible and was used to orientate correctly during cryosectioning. The brain was placed to solidify at −20 °C for 2 h then stored at −80 °C. Coronal mouse brain sections were obtained at 10 µm thickness using a cryostat (ThermoFisher Scientific, San Jose, CA, USA) at −20 °C. The brain tissue sections were adhered to ITO-coated microscope slides and stored at −80 °C until use. A 10-month-old APP/PS1 male mouse brain was processed with the same protocol. For the method optimization experiments, sagittal tissue sections were used instead of coronal tissue sections.

### 2.2. MALDI-MS Spotting Experiments

Five (5) solutions were prepared: DHB (20 mg/mL) in 50% ACN 0.1% TFA, GT (50 mM) in methanol, CMPI (50 mM) in methanol, TEA (50 mM) in methanol, and FA standard (1 mg/mL) in chloroform. For the FA standard, we used FA 16:1, FA 18:1, and FA 20:1. Then, 5 μL of each solution (with the exception of DHB solution) was put into a reaction tube, and the reaction tube was placed on a vortex machine for 10 min. After that process was completed, the contents of the reaction tube were mixed with DHB solution. Next, 1 μL of the resulting mixture was transferred onto the MALDI plate for further MALDI-MS analysis. A Bruker rapifleX MALDI Tissuetyper TOF mass spectrometer (Bruker Scientific, LLC, Bremen, Germany) was used for the MALDI-MS analysis.

The same process was repeated for different concentrations of the FA standard solution. There was a series of diluted FA standard solutions that started from 1 mg/mL and were serially diluted from 5 to 854 times. With that, it was possible to generate a calibration curve for GT-labeled FA standards. A PC 36:2 standard was used as an internal standard for signal normalization.

For the control data in negative ion mode, the FA 16:1 standard was 1 mg/mL, the FA 18:1 standard was 2 mg/mL, and the FA 20:1 standard was 2 mg/mL. Here, 1 μL of FA standard solution was spotted on the MALDI sample plate and mixed with 1 μL of 9-AA solution. The resulting mixture was analyzed via MALDI-MS spotting. This process was repeated for multiple concentrations of the FA standards with 1–128 times serial dilutions. With that, a calibration curve for unlabeled FA standards could be generated. Lyso PG 20:1 was used as an internal standard for signal normalization.

### 2.3. Sample Preparation for MALDI-MS Imaging

The schematic analytical workflow for on-tissue labeling of FAs with the GT derivatization reagent for MALDI-MSI is shown in Figure S1. Three (3) tissue slides were placed in a vacuum desiccator at room temperature for 10 min. Then, the 3 slides were placed in an M5 sprayer (HTX Technologies, Carrboro, NC, USA). GT (10 mM) and CMPI (10 mM) were dissolved in 70% ACN, mixed, and then sprayed onto the tissue slides. The following parameters were set in the M5 sprayer: flow rate of 20 μL/min, tracking space of 2 mm, 30 s drying time between each pass, nozzle temperature set to 35 °C, nozzle nitrogen gas pressure of 10 psi, and moving velocity of the nozzle of 1000 mm/min. There was one parameter that was not kept the same for all 3 tissue slides: the number of passes. One slide had 2 passes, another slide had 4 passes, and another slide had 8 passes. Note that this work on testing different numbers of passes was only for the method optimization experiment; for the WT versus APP/PS1 experiment, only 8 passes were used. Next, the tissue slides were incubated in a sealed chamber with 10% TEA in 70% ACN vapor for 1 h.

After the TEA incubation, the slides were again placed in the M5 sprayer. A control slide without GT was also added to the M5 sprayer. Note that this control slide without GT was only for the method optimization experiment; we did not use a control slide without GT for the WT versus APP/PS1 experiment. CHCA at 5 mg/mL in 70% ACN 0.1% formic acid was sprayed onto the tissue slides. The following parameters were set in the M5 sprayer: flow rate of 50 μL/min, tracking space of 2 mm, 12 passes, 30 s drying time between each pass, nozzle temperature set to 75 °C, nozzle nitrogen gas pressure of 10 psi, and moving velocity of the nozzle of 1000 mm/min. For the WT versus APP/PS1 experiment, the same sample preparation procedure was applied to the WT mouse brain tissue slide and APP/PS1 mouse brain tissue slide.

### 2.4. MALDI-MS Imaging Data Acquisition and Analysis

A Bruker rapifleX MALDI Tissuetyper TOF mass spectrometer (Bruker Scientific, LLC, Bremen, Germany) was used to analyze the tissue slides. For the experiments comparing different amounts of GT sprayed on the tissue sections, the following parameters were used: the raster step size was set at 50 μm, there were 100 shots per pixel, and the sampling rate was 5.00 GS/s. The mass range was set to 200–1000 Da, and the laser energy was set to 32%. External calibration was performed using red phosphorus, as previously described in the literature [30]. Images were visualized using SCiLS Lab version 2023a Pro (Bruker Daltonics, Bremen, Germany), using the total ion count (TIC) for data normalization. The same approach was used for the WT and APP/PS1 tissue slides, only the mass range was set to 300–1000 Da, and the laser energy was set to 50%.

### 2.5. LC-MS/MS Experiments

On-tissue chemical derivatization was performed on a mouse brain tissue section using the procedure described above. After the completion of the on-tissue chemical derivatization, the tissue section was placed in the SepQuant dropletProbe^TM^ system (HTX Technologies, LLC, Chapel Hill, NC, USA). The extraction solvent was 70% ACN with 0.1% formic acid. A probe-to-surface distance of 0.2 mm was found to produce optimal liquid microjunction formation with the high organic content of the extraction solvent. The needle was filled with 2.5 μL of solvent and dispensed 2 μL of this solvent to form a roughly 2.5 mm diameter microjunction. After 2 s, the resulting tissue extract solution was recollected. Three cycles total of microjunction extraction and collection were performed before 2 μL of the needle contents were recollected in Waters LCMS clear glass total recovery vials (Waters Corporation, Milford, MA, USA) prefilled with 10 μL of extraction solvent. The same procedure was repeated in four different spots on the tissue section, yielding four tissue extract samples. The collection vials were stored at −80 °C until LC-MS/MS analysis.

The LC separation was performed on a Waters NanoAcquity UPLC system (Waters, Milford, MA, USA) with a self-fabricated capillary C18 column (20 cm, 75 μm i.d., 2 μm ethylene-bridged hybrid C18 packing material). Mobile phase A was water containing 0.1% formic acid, and mobile phase B was 40:60 IPA/ACN containing 0.1% formic acid. Samples were diluted to 50% ACN 0.1% formic acid with aqueous 0.1% formic acid prior to injection. The flow rate was set at 0.25 μL/min. The following gradient was used: (time, % mobile phase B): (0 min, 20%), (82 min, 95%), (82.5 min, 98%), (142 min 98%), (142.5 min, 20%), and (160 min, 20%).

A Q-Exactive mass spectrometer (Thermo Scientific, Bremen, Germany) was used for MS detection. The following mass spectrometer parameters were used for data acquisition: positive ion mode was used for ionization, the S-lens radio frequency (RF) level was set to 55, and the capillary temperature was 275 °C. Full MS scans were acquired at *m*/*z* 300–1000 with a resolving power of 70 K. For full MS scans, a maximum injection time of 250 ms and an automatic gain control (AGC) target value of 1e6 were applied. MS/MS scans were acquired with a resolving power of 35 k. An inclusion list containing *m*/*z* values of FA derivatives was used to trigger MS/MS with ±25 ppm mass tolerance. The top 15 ions were selected with a 1.2 *m*/*z* isolation window and fragmented with high-energy collision dissociation (HCD) at a stepped normalized collision energy (NCE) of 25, 30. The maximum injection time was set at 100 ms, and the automatic gain control (AGC) target value was set at 2 × 10^5^.

## 3. Results and Discussion

### 3.1. Improvement in Detection Sensitivity in Positive Mode with GT Reaction

In the absence of chemical derivatization, FAs ionize preferentially in negative ion mode. There was a need to see whether using the GT reaction to detect FAs in positive ion mode would lead to better detection sensitivity than negative ion mode would allow for. To answer this question, commercial FA standards were used, namely FA 16:1, FA 18:1, and FA 20:1. Each FA standard was prepared at different concentrations so that a limit of quantification (LOQ) could be determined. This was done both in the negative ion mode without the GT reaction and in the positive ion mode with the GT reaction. Lyso PG 20:1 and PC 36:2 were used as internal standards in negative mode and positive mode, respectively.

**Figure 1:**
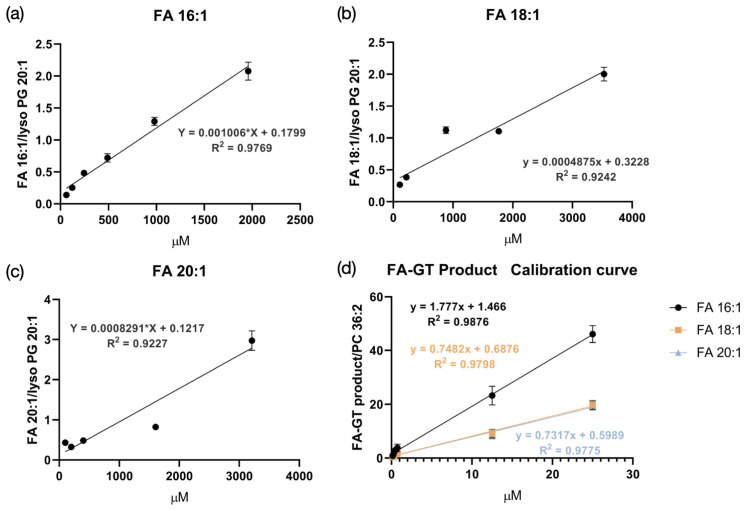
Calibration curves in negative mode and in positive mode with Girard’s Reagent T (GT) reaction. (**a**–**c**) Calibration curves for fatty acid (FA) 16:1, FA 18:1, and FA 20:1 in negative mode. (**d**) Calibration curves for the same molecules in positive mode with GT labeling. The limit of quantification (LOQ) was 61.2 μM, 110.3 μM, and 100.4 μM in negative ion mode for FA 16:1, FA 18:1, and FA 20:1, respectively, while the LOQ was 146.3 nM for all 3 FAs in positive ion mode.

The results of this experiment are shown in Figure 1 and Tables S1–S6. While the negative mode experiments were performed with 1–128 times serial dilutions, Tables S1–S3 only show data for 1–32 times serial dilutions. The reason for this is that the unlabeled FA standards were not detected in negative mode when the dilution factor was larger than 32. In negative ion mode, the LOQ for FA 18:1 was 110.3 μM, and the LOQ for FA 20:1 was 100.4 μM. However, with the GT reaction, the LOQ for those same FAs decreased by a factor of 1000 to 146.3 nM; this indicates a 1000-fold improvement in the detection sensitivity. Clearly, the GT derivatization method is an improvement upon the conventional methodology used to detect FAs.

It is worth mentioning that these data were collected via MALDI-MS spotting. Unlike LC-MS data, MALDI-based quantification suffers from variation [31,32]. Due to variability in signal intensity during MALDI spotting, the quantification linearity for FAs in this experiment was suboptimal. We include the detailed data points in the Supplementary Materials as Tables S1–S6, but in this study, we primarily focus on developing a chemical derivatization method applicable to MSI to enable the visualization of FA distribution in tissue samples.

### 3.2. MALDI-MS Imaging of Mouse Brain Tissue Sections Using the GT Method

MALDI-MS imaging was performed on multiple tissue sections, with the method on the automated sprayer being different for each tissue section. The number of passes (that is, the number of layers) of GT that was deposited onto the tissue section differed from one section to another. Eight passes of GT, four passes of GT, two passes of GT, and zero passes of GT were all tested.

**Figure 2:**
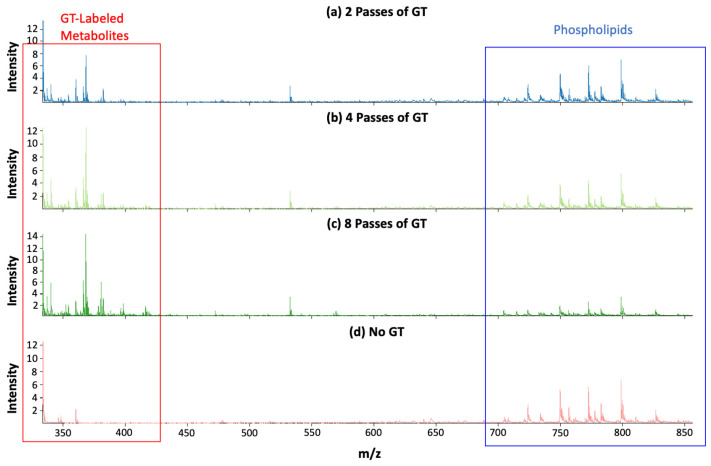
Normalized mass spectra for (**a**) dataset with 2 passes of GT, (**b**) dataset with 4 passes of GT, (**c**) dataset with 8 passes of GT, and (**d**) dataset with no GT. The GT-labeled metabolites *m*/*z* range and phospholipids *m*/*z* range are indicated with a red framed box and blue framed box in the figure, respectively.

It was found that zero passes of GT gave the highest signal in the phospholipid *m*/*z* range, while eight passes of GT gave the lowest signal in the phospholipid *m*/*z* range. The converse was true for the GT-labeled metabolites *m*/*z* range. There were many peaks in the GT-labeled metabolites *m*/*z* range that were only seen with GT derivatization, as shown in Figure 2. One more subtle observation about the mass spectra (in Figure 2) is that *m*/*z* 390–420 contains peaks that can only be seen with eight passes of GT; those same peaks cannot be seen with four passes of GT or two passes of GT.

**Figure 3:**
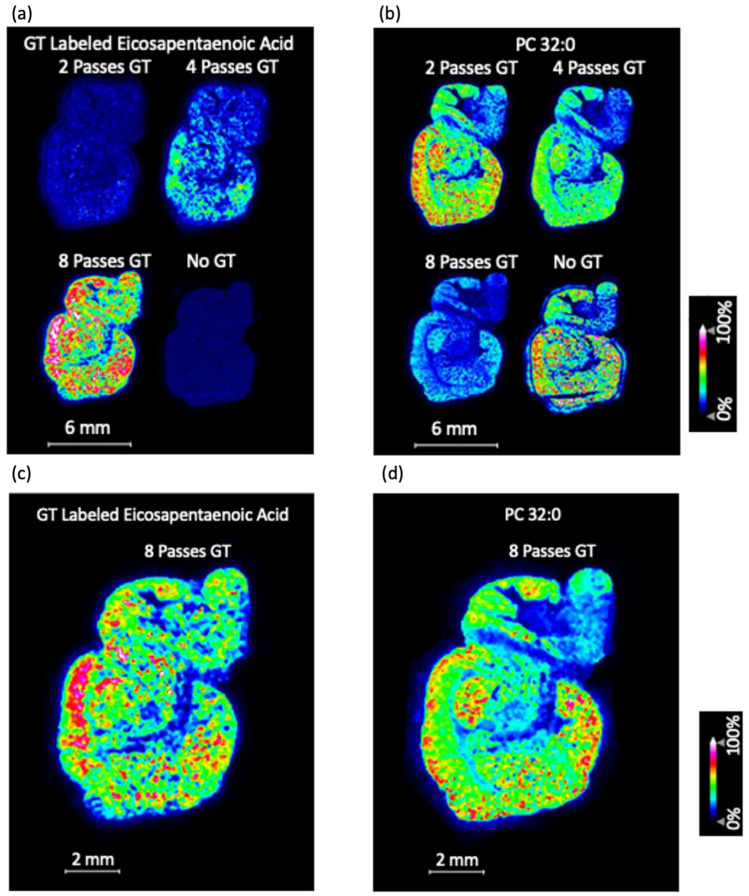
Number of passes for GT and its effect on FA signals and phospholipid signals. (**a**) Mass spectrometry (MS) images for GT-labeled eicosapentaenoic acid (*m*/*z* 416.339) with 2 passes of GT (top left), 4 passes of GT (top right), 8 passes of GT (bottom left), and no GT (bottom right). (**b**) [Phospatidylcholine 32:0 + K]^+^ ([PC 32:0 + K]^+^, *m*/*z* 772.539) with 2 passes of GT (top left), 4 passes of GT (top right), 8 passes of GT (bottom left), and no GT (bottom right). (**c**) GT-labeled eicosapentaenoic acid (*m*/*z* 416.377) with 8 passes of GT. (**d**) [PC 32:0 + K]^+^ (*m*/*z* 772.451) with 8 passes of GT.

Representative MS images can be found in Figure 3. Figure 3a clearly demonstrates that eight passes of GT give the highest signal for GT-labeled FAs, and a representative FA image with eight passes of GT is shown in Figure 3c. Figure 3b clearly demonstrates that eight passes of GT give the lowest signal for phospholipids. However, the phospholipid images with 8 passes of GT look weak only relative to phospholipid images with less GT. As can be seen in Figure 3d, there is still a noticeable signal for the phospholipid image with eight passes of GT when it is not being compared to the tissue sections with less GT. Consequently, the eight-pass GT method can be used for phospholipids. The phospholipid signal with eight passes of GT may not be as good as it would be with less GT, but if the main priority is to obtain a high signal for FAs, eight passes of GT is optimal. Based on this information, we selected eight passes as the amount of GT to spray over the WT and APP/PS1 mouse brain tissue sections.

The inverse relationship between the amount of GT and the phospholipid signal invites the following question: why does this inverse relationship exist? One possible explanation is charge competition between excess derivatization reagent and the phospholipids; an excess of derivatization reagent may compete with phospholipids for ionization, thereby reducing phospholipid signal intensities [24]. Another possible explanation is that increasing the number of GT passes extends the total spraying time at room temperature, which can lead to phospholipid hydrolysis. Notably, eight passes of GT require about 23 min of spraying, whereas only two passes take significantly less time. This shorter exposure likely mitigates hydrolysis, which aligns with the stronger phospholipid signals seen at two passes. In practice, both mechanisms—charge competition and time-dependent hydrolysis—could collectively account for the lower phospholipid signals observed as eight passes of GT are applied.

**Figure 4:**
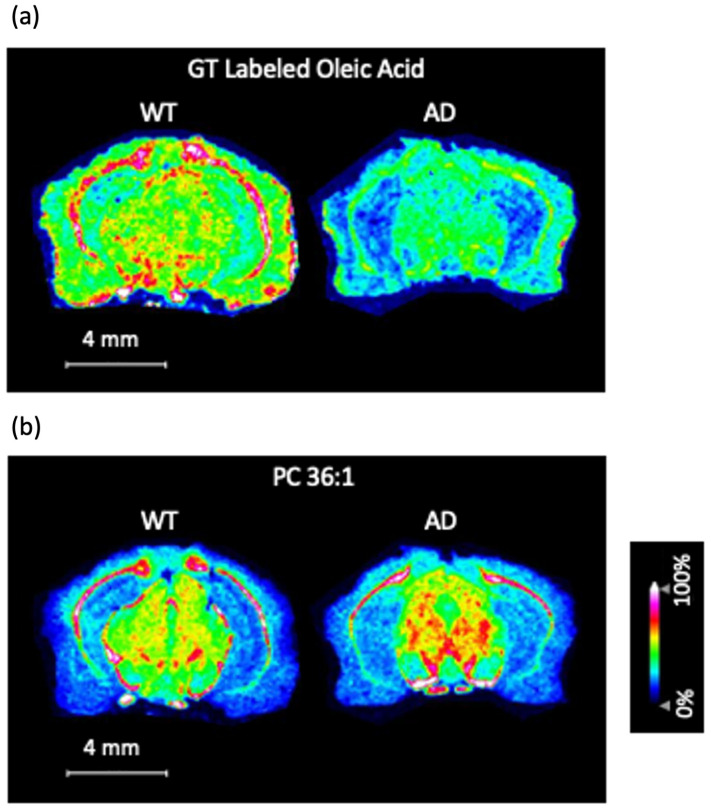
Wild-type (WT) vs. Alzheimer’s disease (AD) mouse model MS images for (**a**) GT-labeled oleic acid (*m*/*z* 396.421) and (**b**) [PC 36:1 + K]^+^ (*m*/*z* 826.568). In this case, the AD mouse model is amyloid precursor protein/presenelin 1 (APP/PS1). Please refer to the mouse brain atlas coronal tissue section figure at bregma −0.82 mm to see the brain regions. (Bregma −0.82 mm is the depth used in our experiment.) Note that the brain region with the label “cc” is the corpus callosum. The link to the atlas figure can be found here: https://labs.gaidi.ca/mouse-brain-atlas/?ml=&ap=-0.82&dv= (accessed on 26 December 2024).

After repeating the same procedure, MS images for GT-labeled FAs were collected from WT and APP/PS1 mouse brain tissue sections. It was observed that oleic acid was depleted in the APP/PS1 corpus callosum (Figure 4a). The data were consistent across three technical replicates (Figure S2), thus demonstrating the reproducibility and utility of the GT method.

Not only is the GT method highly reproducible, but it has a major advantage over negative mode FA imaging: there is no issue with undesired lipid fragmentation. In negative mode FA imaging, high laser energy leads to the fragmentation of larger lipids, thus bringing forth FA fragments. The FA fragments are indistinguishable from the FAs originally in the tissue, thus making the FA signals questionable [33]. The GT method bypasses this issue, making the FAs originally in the tissue distinguishable from FA fragments.

The GT method also provides biologically meaningful results. To the best of our knowledge, no other research group has observed a decrease in the levels of free oleic acid in the APP/PS1 corpus callosum. However, our data are still consistent with the existing literature. It was observed by Khamidova et al. [34] that free oleic acid is present at higher levels in the white matter (when compared to other free FAs); given the established knowledge that the corpus callosum is a white matter tract, it would logically follow that free oleic acid should be present at high levels in the corpus callosum. This is reinforced by the existing literature. Given the established knowledge that myelin is an integral component of white matter, it is important to note that the literature provides ample evidence for oleic acid being highly abundant in myelin [13]. Oleic acid plays an important role in myelin, which is to synthesize myelin phospholipids [35].

If free oleic acid is missing within the APP/PS1 corpus callosum, that would mean that some important myelin phospholipids cannot be synthesized, thus harming myelin function within the corpus callosum. There is evidence in the literature that myelin function plays a crucial role in memory consolidation and cognition [36]. Consequently, the results we reported here have implications in AD pathogenesis.

The free oleic acid data led to an interesting question. There is an important myelin phospholipid species that contains oleic acid: PC 36:1 [37]. Multiple MS imaging studies have shown that [PC 36:1 + K]^+^ is distributed in the white matter [38,39], and a study from Trépanier et al. has shown that PC 36:1 is downregulated in the corpus callosum for a mouse model of multiple sclerosis [37]. Given the free oleic acid data that we obtained, it was of interest to see if there is an observed depletion of [PC 36:1 + K]^+^ in the corpus callosum to match the findings of Trépanier et al. for the multiple sclerosis mouse model. We did not observe the depletion of [PC 36:1 + K]^+^ in the APP/PS1 corpus callosum (Figure 4b).

Our data for [PC 36:1 + K]^+^ illustrate an advantage of the GT method. When the question arose about PC 36:1, there was no need to perform a separate experiment to answer it. The PC 36:1 data were collected in the same imaging run as the GT labeled oleic acid data. Had we performed imaging of FAs in the negative ion mode, we would need to perform a separate experiment in the positive ion mode to answer the question about PC 36:1. It is also important to note that the quality of the PC data is not significantly compromised by the GT method. Our [PC 36:1 + K]^+^ images show distribution in the white matter, which is in agreement with the previous literature [38,39]. Furthermore, our [PC 36:1 + K]^+^ images look consistent across three technical replicates, at least with respect to the corpus callosum (Figure S3); this clearly demonstrates the reproducibility of the data.

One limitation of our experimental method is that it does not distinguish between oleic acid (also known as FA 18:1n-9) and its double-bond-position isomers; oleic acid has multiple double-bond-position isomers, including FA 18:1n-6, FA 18:1n-7, FA 18:1n-8, and FA 18:1n-11. In the future, we can apply our previously published epoxidation method that used peracetic acid to distinguish between FA double-bond-position isomers [40]. The peracetic acid method can be combined with the GT method, yielding GT-labeled epoxy-FAs that can be detected in the positive ion mode. With this strategy, GT-labeled epoxy oleic acid can be distinguished from GT-labeled epoxy FA 18:1n-6, GT-labeled epoxy FA 18:1n-7, and so on.

Similarly, PC 36:1 has double-bond-position isomers [37]. For example, PC 18:0/18:1n-9 and PC 18:0/18:1n-7 cannot be distinguished through our experimental method; this matters because PC 18:0/18:1n-9 is the molecule that specifically contains oleic acid. The PC isomer issue can be addressed in the same way as the FA isomer issue: by combining the peracetic acid epoxidation method with the GT method and performing MS/MS imaging. Through this approach, both GT-labeled epoxy oleic acid and epoxy PC 18:0/18:1n-9 can be detected in the positive ion mode. The isomers of both GT-labeled FAs and PCs can be detected simultaneously.

Since our current methodology does not distinguish between oleic acid and its isomers, we must exercise caution when naming the molecule in Figure 4a as oleic acid. In actuality, we have not definitively proven that this molecule is oleic acid; it could be a double-bond-position isomer of oleic acid. The molecule in question may be oleic acid, but this needs to be confirmed. In order to confirm the identity of the molecule in question, it will be of high importance to conduct future studies that combine the epoxidation method with the GT method.

It is also worth noting that the data presented here represent a proof-of-principle application of the in situ GT derivatization method. Another future direction is to perform studies on a larger cohort of mice to confirm and validate that oleic acid is indeed downregulated in the APP/PS1 corpus callosum. Not only is it beneficial to increase the sample size, but it is also beneficial to include additional mouse models of AD in future studies. If similar findings are seen in other mouse models of AD, more definitive and generalized biological conclusions can be reached.

Another future direction is to apply the GT method to other biological tissues and diseases for expanded scope and impact of the methodology. This idea has potential, as FAs have been shown to be relevant to type 2 diabetes [41], cancer [42], and Parkinson’s disease [43]. However, care must be taken to make sure the biological tissue of interest is compatible with the GT method. It is important to use fresh frozen tissue rather than formalin-fixed, paraffin-embedded (FFPE) tissue; the preparation of FFPE tissue leads to loss of FAs. This is particularly important to remember when working with clinical cancer samples, as most clinical samples are FFPE tissue.

### 3.3. LC-MS/MS Analysis of GT-Labeled FA Extracts

We conducted LC-MS/MS analysis to verify the presence of chemically derivatized FAs following our established protocol for on-tissue chemical derivatization of mouse brain tissue sections. Localized extractions were carried out at four distinct spots on each section, yielding four separate tissue extract solutions. These were then analyzed using LC-MS/MS, as detailed in Section 2. We successfully obtained MS/MS spectra of eight specific GT-labeled FAs across all extraction spots, including C16:1, C16:0, C18:1, C18:0, C20:5, C20:4, C20:1, and C22:6, as presented in Figures S4–S11. The comprehensive spectral data confirmed the presence of these target GT-labeled FAs, validating that our derivatization method effectively labels FAs in situ within mouse brain tissue. Detailed analytical data for each spot, including the retention times, signal intensities at various locations, and mass errors, are systematically documented in Tables S7–S11. This dataset supports the reproducibility and reliability of our on-tissue GT derivatization technique.

## 4. Conclusions

We developed a novel on-tissue chemical derivatization method for MALDI-MS imaging of FAs in the mouse brain, using GT as the derivatization reagent. It is important to note that this method enables a 1000-fold improvement in detection sensitivity when compared to conventional negative ion mode analysis of FAs. Given this information, the method was used to perform imaging of FAs in WT versus APP/PS1 AD mouse brain tissue sections. It was observed that there was depletion of oleic acid in the APP/PS1 corpus callosum. Notably, our method for FA imaging does not significantly compromise the quality of the phospholipid imaging data. Furthermore, MS/MS spectra were collected from four different spots on a mouse brain tissue section via LC-MS/MS experiments to confirm the identities of GT-labeled FAs in mouse brain tissue. This method can be broadly transferred to the FA mapping of other biological tissues to enable the simultaneous mapping of FAs and phospholipids in positive ion mode with enhanced sensitivity and throughput.

## Data Availability

The raw data supporting the conclusions of this article will be made available by the authors upon request.

## Supplementary Materials

The following supporting information can be downloaded at https://www.mdpi.com/article/10.3390/biom15030366/s1: Figure S1: Schematic analytical workflow for on-tissue labeling of fatty acids (FAs) with the Girard’s Reagent T (GT) derivatization reagent for mass spectrometry imaging (MSI); Table S1: Data used for calibration curve of FA 16:1 standard in negative mode; Table S2: Data used for calibration curve of FA 18:1 standard in negative mode; Table S3: Data used for calibration curve of FA 20:1 standard in negative mode; Table S4: Data used for calibration curve of GT-labeled FA 16:1 standard in positive mode; Table S5: Data used for calibration curve of GT-labeled FA 18:1 standard in positive mode; Table S6: Data used for calibration curve of GT-labeled FA 20:1 standard in positive mode; Figure S2: Technical replicates for GT-labeled oleic acid in wild-type (WT) vs. Alzheimer’s disease (AD) mouse model MS imaging data; Figure S3: Technical replicates for [Phosphatidylcholine 36:1 + K]^+^ ([PC 36:1 + K]^+^) in WT vs. AD mouse model MS imaging data; Figure S4: MS/MS spectrum for GT-labeled FA 16:1 from tissue extract solution; Figure S5: MS/MS spectrum for GT-labeled FA 16:0 from tissue extract solution; Figure S6: MS/MS spectrum for GT-labeled FA 18:1 from tissue extract solution; Figure S7: MS/MS spectrum for GT-labeled FA 18:0 from tissue extract solution; Figure S8: MS/MS spectrum for GT-labeled FA 20:5 from tissue extract solution; Figure S9: MS/MS spectrum for GT-labeled FA 20:4 from tissue extract solution; Figure S10: MS/MS spectrum for GT-labeled FA 20:1 from tissue extract solution; Figure S11: MS/MS spectrum for GT-labeled FA 22:6 from tissue extract solution; Table S7: Retention times for different GT-labeled FAs; Table S8: Signal intensities and mass errors for different GT-labeled FAs at extraction spot 1; Table S9: Signal intensities and mass errors for different GT-labeled FAs at extraction spot 2; Table S10: Signal intensities and mass errors for different GT-labeled FAs at extraction spot 3; Table S11: Signal intensities and mass errors for different GT-labeled FAs at extraction spot 4.
