# Supplementary material for: On-Tissue Chemical Derivatization for Mass Spectrometry Imaging of Fatty Acids with Enhanced Detection Sensitivity
Source: Biomolecules. 2025 Mar 3;15(3):366. doi: 10.3390/biom15030366 (PMC11940502; doi:10.3390/biom15030366)
Supplement: Supplementary file 1 [file biomolecules-15-00366-s001.zip › biomolecules-3422678-supplementary.pdf]

Supplementary Information for

On-Tissue Chemical Derivatization for Mass Spectrometry Imaging of Fatty Acids with Enhanced Detection Sensitivity

Malik Ebbini [1,†], Zicong Wang [1,†], Hua Zhang [1,*], Kelly H. Lu [2], Penghsuan Huang [2], Cameron J. Kaminsky [2], Luigi Puglielli [3,4,5] and Lingjun Li [1,2,6,7,*]

† These authors contributed equally to this work

*Lingjun Li and Hua Zhang are the corresponding author

[1]School of Pharmacy, University of Wisconsin-Madison, Madison, Wisconsin 53705, United States

[2]Department of Chemistry, University of Wisconsin-Madison, Madison, Wisconsin 53706, United States

[3]Department of Medicine, University of Wisconsin-Madison, Madison, Wisconsin 53705, United States

[4]Waisman Center, University of Wisconsin-Madison, Madison, Wisconsin 53705, United States

[5]Geriatric Research Education Clinical Center, Veterans Affairs Medical Center, Madison, Wisconsin 53705, United States

[6]Lachman Institute for Pharmaceutical Development, School of Pharmacy, University of Wisconsin-Madison, Madison, Wisconsin 53705, United States

[7]Wisconsin Center for NanoBioSystems, School of Pharmacy, University of Wisconsin-Madison, Madison, Wisconsin 53705, United States

Table of Contents

Figure S1. Schematic analytical workflow for on-tissue labeling of fatty acids (FA) with the Girard's Reagent T (GT) derivatization for mass spectrometry imaging (MSI)

Table S1. Data used for calibration curve of FA 16:1 standard in the negative ion mode

Table S2. Data used for calibration curve of FA 18:1 standard in the negative ion mode

Table S3. Data used for calibration curve of FA 20:1 standard in the negative ion mode

Table S4. Data used for calibration curve of GT labeled FA 16:1 standard in the positive ion mode

Table S5. Data used for calibration curve of GT labeled FA 18:1 standard in the positive ion mode

Table S6. Data used for calibration curve of GT labeled FA 20:1 standard in the positive ion mode

Figure S2. Technical replicates for GT labeled Oleic Acid in Wild-Type (WT) *vs.* Alzheimer's Disease (AD) Mouse Model MS Imaging

Figure S3. Technical replicates for [Phosphatidylcholine 36:1 + K]$^+$ ([PC 36:1+K]$^+$) in WT vs. AD Mouse Model MS imaging

Figure S4. MS/MS spectrum for GT labeled FA 16:1 from tissue extract solution

Figure S5. MS/MS spectrum for GT labeled FA 16:0 from tissue extract solution

Figure S6. MS/MS spectrum for GT labeled FA 18:1 from tissue extract solution

Figure S7. MS/MS spectrum for GT labeled FA 18:0 from tissue extract solution

Figure S8. MS/MS spectrum for GT labeled FA 20:5 from tissue extract solution

Figure S9. MS/MS spectrum for GT labeled FA 20:4 from tissue extract solution

Figure S10. MS/MS spectrum for GT labeled FA 20:1 from tissue extract solution

Figure S11. MS/MS spectrum for GT labeled FA 22:6 from tissue extract solution

Table S7. Retention times for different GT labeled FAs

Table S8. Signal intensities and mass errors for different GT labeled FAs at extraction spot 1

Table S9. Signal intensities and mass errors for different GT labeled FAs at extraction spot 2

Table S10. Signal intensities and mass errors for different GT labeled FAs at extraction spot 3

Table S11. Signal intensities and mass errors for different GT labeled FAs at extraction spot 4

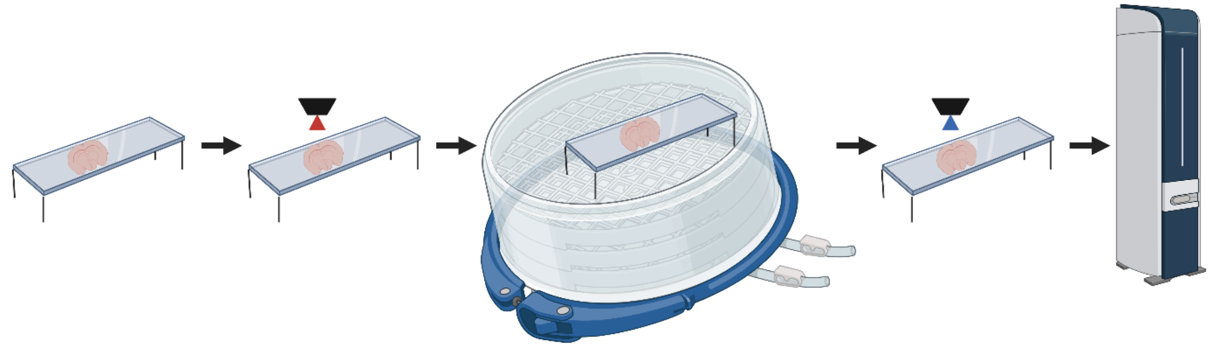

Figure S1. Schematic analytical workflow for on-tissue labeling of fatty acids (FA) with the Girard's Reagent T (GT) derivatization reagent for mass spectrometry imaging (MSI). In this workflow, we used 2-chloro-1-methylpyridinium iodide (CMPI) as the coupling reagent, triethylamine (TEA) to provide a basic environment for the reaction, and matrix-assisted laser desorption/ionization (MALDI) as the ionization method.

Note On Calibration Curves

It is important to reiterate that the calibration curve data was collected through MALDI spotting. MALDI spotting semi-quantification often suffers from spot-to-spot variation that results from several factors (contributing factors include spot crystallization, matrix-to-analyte ratio, hot-spot effect, variation in ionization efficiency compared to LC-MS, and ion suppression due to lack of separation) [31,32]. Although we used internal standards for normalization and increased the number of replicates to minimize variation, we still observed unavoidable variations in quantification. Due to this inherent limitation of MALDI spotting, we identified two data points that behaved as outliers. These outlier points are indicated in **Tables S2-S3** and were excluded from our final analysis to improve the accuracy of the linear regression results.

It is also important to note that the focus of this manuscript is not quantitation; the focus of this manuscript is the development of a novel on-tissue chemical derivatization method for MS imaging of FAs in tissue samples. Even if the calibration curves do not have perfect linearity, the calibration curves still demonstrate the ability to detect the GT labeled FAs at much lower concentrations compared to the unlabeled FAs under negative ion mode.

Table S1. Data used for calibration curve of FA 16:1 standard in the negative ion mode. The limit of quantification (LOQ) is 61.2 μM

Dilute By X	Conc. (μM)	Sig. Intensity for FA 16:1 in Negative Mode (Five Replicates)					Average (n=5)	SD	RSD (%)
1x	1959.0	2.0291	2.0473	2.1279	2.2804	1.8982	2.0766	0.1406	6.77
2x	979.5	1.2894	1.2024	1.3289	1.3694	1.2608	1.2902	0.0639	4.95
4x	489.7	0.6321	0.7579	0.6723	0.7478	0.7877	0.7196	0.0648	9.00
8x	244.9	0.4885	0.4434	0.4549	0.4840	0.5417	0.4825	0.0382	7.91
16x	122.4	0.2607	0.2553	0.2535	0.2492	0.2515	0.2540	0.0043	1.71
32x	61.2	0.1469	0.1324	0.1346	0.1333	0.1418	0.1378	0.0063	4.56

Table S2. Data used for calibration curve of FA 18:1 standard in the negative ion mode. The LOQ is 110.3 μM. Without the data point at 8x, R^2 is 0.92. With that point, R^2 is 0.02.

Dilute By X	Conc. (μM)	Sig. Intensity for FA 18:1 in Negative Mode (Five Replicates)					Average (n=5)	SD	RSD (%)
1x	3530.3	2.0102	1.9634	2.1124	2.0753	1.8418	2.0006	0.1058	5.29
2x	1765.1	1.0891	1.0857	1.1100	1.1386	1.1027	1.1052	0.0211	1.91
4x	882.6	1.1784	1.1366	1.0817	1.1620	1.0538	1.1225	0.0531	4.73
~~*8x~~	~~441.3~~	~~5.2917~~	~~5.5676~~	~~0.1162~~	~~0.1475~~	~~5.0152~~	~~4.3263~~	~~2.6276~~	~~60.74~~
*16x	220.6	0.3885	0.3907	0.3712	0.3713	0.3583	0.3852	0.0207	5.37
*32x	110.3	0.2873	0.2677	0.2659	0.2682	0.2679	0.2667	0.0102	3.81

Table S3. Data used for calibration curve of FA 20:1 standard in the negative ion mode. The LOQ is 100.4 μM. Without the data point at 4x, R^2 is 0.92. With that point, R^2 is 0.02.

Dilute By X	Conc. (μM)	Sig. Intensity for FA 20:1 in Negative Mode (Five Replicates)					Average (n=5)	SD	RSD (%)
*1x	3212.4	3.3498	3.1018	2.7296	2.6285	3.0514	2.9724	0.2424	8.16
2x	1606.2	0.7720	0.8739	0.8711	0.7998	0.7854	0.8204	0.0486	5.92
~~*4x~~	~~803.1~~	~~7.7314~~	~~7.9941~~	~~7.8042~~	~~10.8171~~	~~10.4746~~	~~9.7313~~	~~1.6448~~	~~16.90~~
*8x	401.5	0.5695	0.5769	0.4788	0.4462	0.4308	0.4852	0.0586	12.07
*16x	200.8	0.3005	0.3167	0.3028	0.3132	0.3211	0.3236	0.0243	7.50
32x	100.4	0.4401	0.4117	0.4243	0.4404	0.4458	0.4325	0.0141	3.26

The starred rows have 8 replicates, not 5. For those rows, the extra 3 replicates are shown below:
FA 18:1 8x – 6.4581; 6.0021; 6.0120
FA 18:1 16x – 0.4256; 0.3780; 0.3979
FA 18:1 32x – 0.2671; 0.2523; 0.2574
FA 20:1 1x – 2.8398; 2.8937; 3.1845
FA 20:1 4x – 12.0318; 10.5492; 10.4479
FA 20:1 8x – 0.4229; 0.4770; 0.4796
FA 20:1 16x – 0.3772; 0.3344; 0.3231

Table S4. Data used for calibration curve of GT labeled FA 16:1 standard in the positive ion mode. The LOQ is 146.3 nM

Dilute by X	Conc. (nM)	Sig. Intensity for FA 16:1 in Positive Mode (Five Replicates)					Average (n=5)	SD	RSD (%)
5x	25000.0	48.1315	49.8214	44.7117	41.6365	46.1663	46.0935	3.1556	6.85
10x	12500.0	27.7593	21.1203	21.6752	19.6379	25.9151	23.2216	3.4459	14.84
112.5x	1111.1	3.9560	3.5810	5.7675	3.4485	6.1450	4.5796	1.2774	27.89
168x	740.7	4.6663	2.9175	1.7346	4.9399	4.6847	3.7886	1.4032	37.04
253x	493.8	2.8838	2.7115	2.9430	3.3052	2.9578	2.9603	0.2162	7.30
379x	329.2	2.9737	1.7559	1.9914	2.2430	2.1607	2.2250	0.4582	20.59
569x	219.5	1.2789	1.0688	1.0589	1.3896	1.2525	1.2097	0.1428	11.81
854x	146.3	0.8233	0.9777	0.9049	0.7714	0.7304	0.8415	0.1002	11.91

Table S5. Data used for calibration curve of GT labeled FA 18:1 standard in the positive ion mode. The LOQ is 146.3 nM

Dilute by X	Conc. (nM)	Sig. Intensity for FA 18:1 in Positive Mode (Five Replicates)					Average (n=5)	SD	RSD (%)
5x	25000.0	20.3980	21.8148	19.2644	17.3609	20.1250	19.7926	1.6400	8.29
10x	12500.0	11.5646	8.4648	8.0609	7.9644	9.9212	9.1952	1.5392	16.74
112.5x	1111.1	1.1008	1.4563	1.5024	1.5543	1.4551	1.4138	0.1796	12.71
168x	740.7	2.2123	1.2294	0.7066	2.6817	2.1027	1.7865	0.7999	44.77
253x	493.8	1.8302	1.5517	1.4196	1.3002	1.7926	1.5789	0.2305	14.60
379x	329.2	1.6168	0.7393	0.9957	0.9691	1.1228	1.0887	0.3260	29.94
569x	219.5	0.5367	0.4044	0.3832	0.4903	0.4835	0.4596	0.0639	13.91
854x	146.3	0.3926	0.4980	0.4532	0.3446	0.3780	0.4133	0.0616	14.90

Table S6. Data used for calibration curve of GT labeled FA 20:1 standard in the positive ion mode. The LOQ is 146.3 nM

Dilute by X	Conc. (nM)	Sig. Intensity for FA 20:1 in Positive Mode (Five Replicates)					Average (n=5)	SD	RSD (%)
5x	25000.0	19.0918	21.4807	18.0153	18.1412	20.6762	19.4810	1.5429	7.92
10x	12500.0	10.7122	8.0450	7.4753	8.8982	7.4317	8.5125	1.3647	16.03
112.5x	1111.1	1.6224	1.5116	1.4373	1.3904	2.1412	1.6206	0.3039	18.76
168x	740.7	2.0568	2.7348	1.1979	0.7396	1.9174	1.7293	0.7771	44.94
253x	493.8	2.1691	1.1422	1.3901	1.4000	1.5627	1.5328	0.3861	25.19
379x	329.2	1.4438	0.7426	0.9856	0.9862	1.0316	1.0380	0.2537	24.44
569x	219.5	0.4288	0.3315	0.3282	0.3725	0.3824	0.3687	0.0414	11.22
854x	146.3	0.3382	0.4530	0.4083	0.3365	0.3641	0.3800	0.0500	13.17

(a)

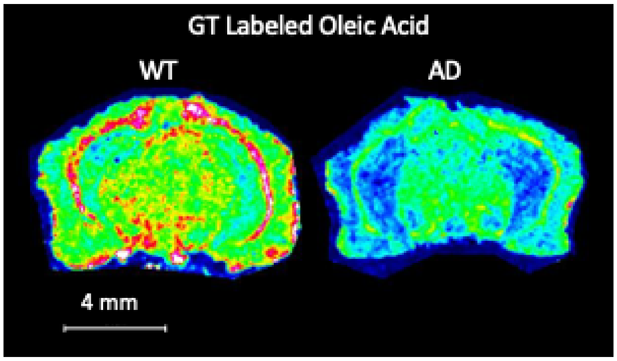

(b)

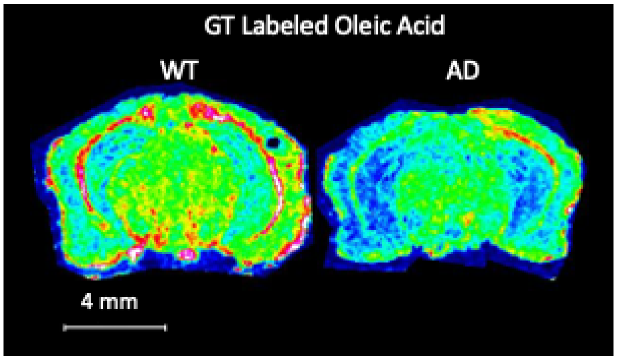

(c)

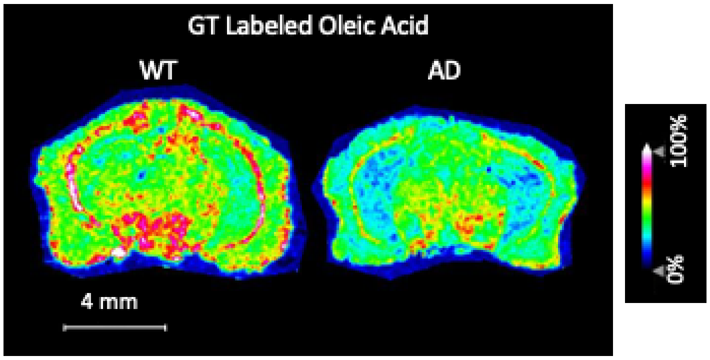

Figure S2. (a) MS images of GT labeled oleic acid in Wild-Type (WT) vs. Alzheimer's Disease (AD) Mouse Model replicate 1 (*m/z* 396.421). (b) MS images of GT labeled oleic acid in WT vs. AD Mouse Model replicate 2 (*m/z* 396.421). (c) MS images of GT labeled oleic acid in WT vs. AD mouse model replicate 3 (*m/z* 396.423). In this case, the AD mouse model we used was Amyloid Precursor Protein/Presenilin 1 (APP/PS1).

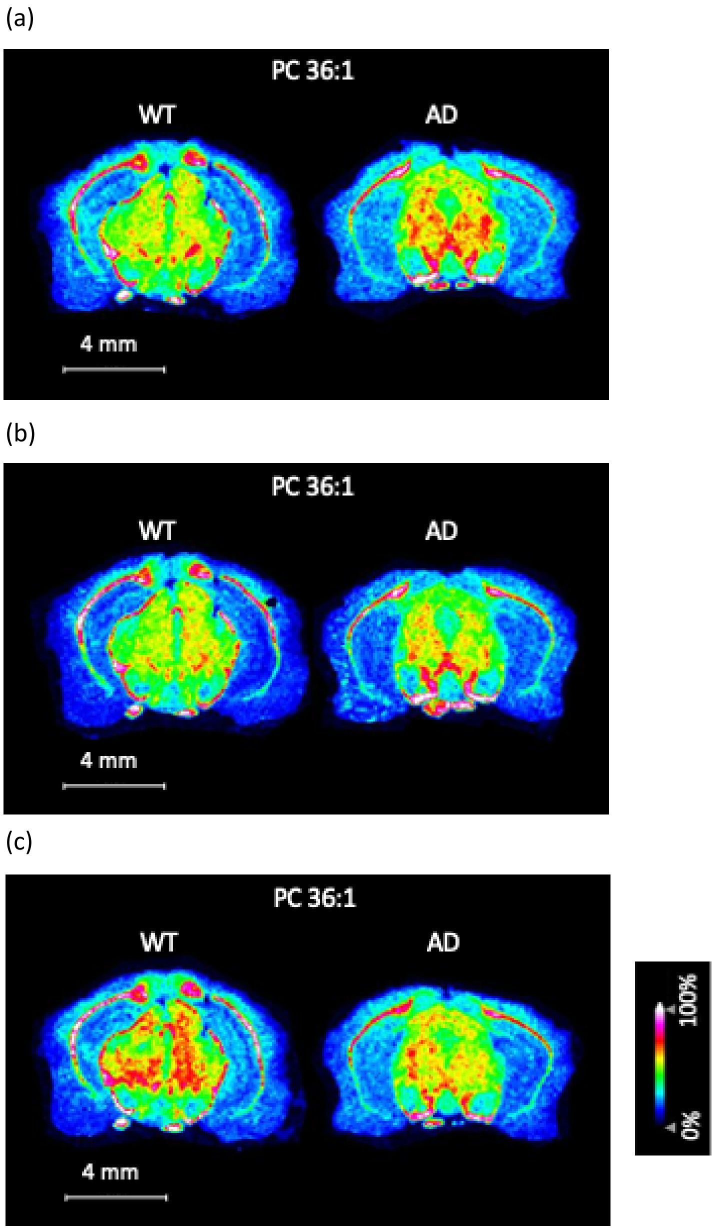

Figure S3. (a) MS images of [Phosphatidylcholine 36:1 + K]$^+$ ([PC 36:1 + K]$^+$) in WT vs. AD Mouse Model replicate 1 (*m/z* 826.568). (b) MS images of [PC 36:1 + K]$^+$ in WT vs. AD Mouse Model replicate 2 (*m/z* 826.615). (c) MS images of [PC 36:1 + K]$^+$ in WT vs. AD Mouse Model replicate 3 (*m/z* 826.592). In this case, the AD mouse model we used was Amyloid Precursor Protein/Presenilin 1 (APP/PS1).

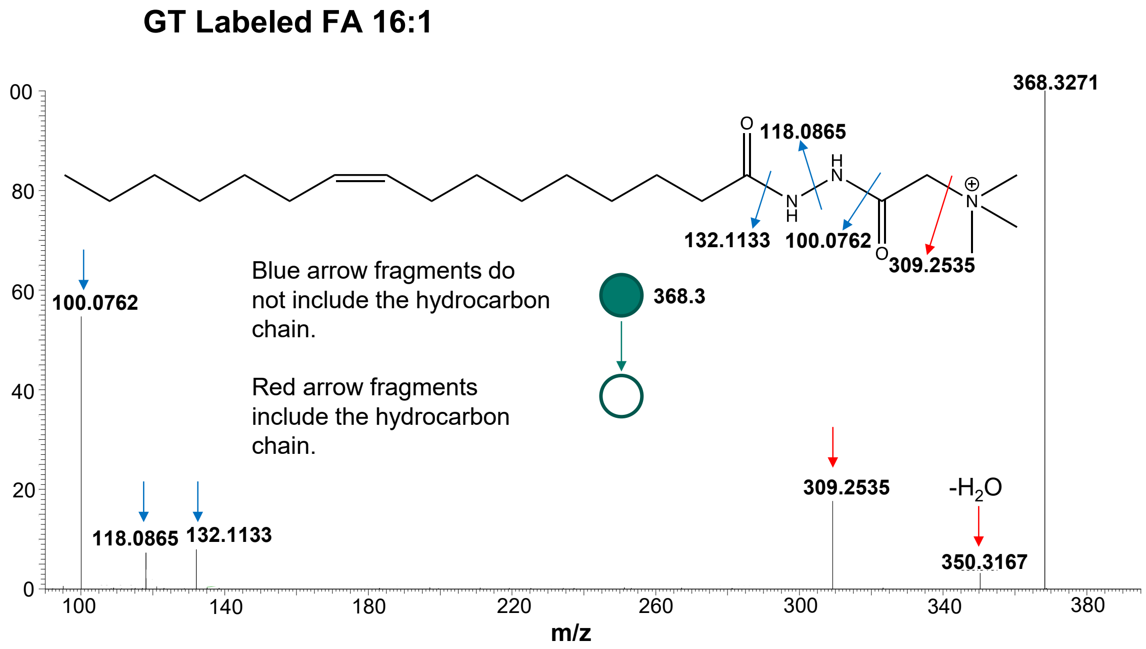

Figure S4. MS/MS spectrum of GT labeled FA 16:1 from tissue extract solution

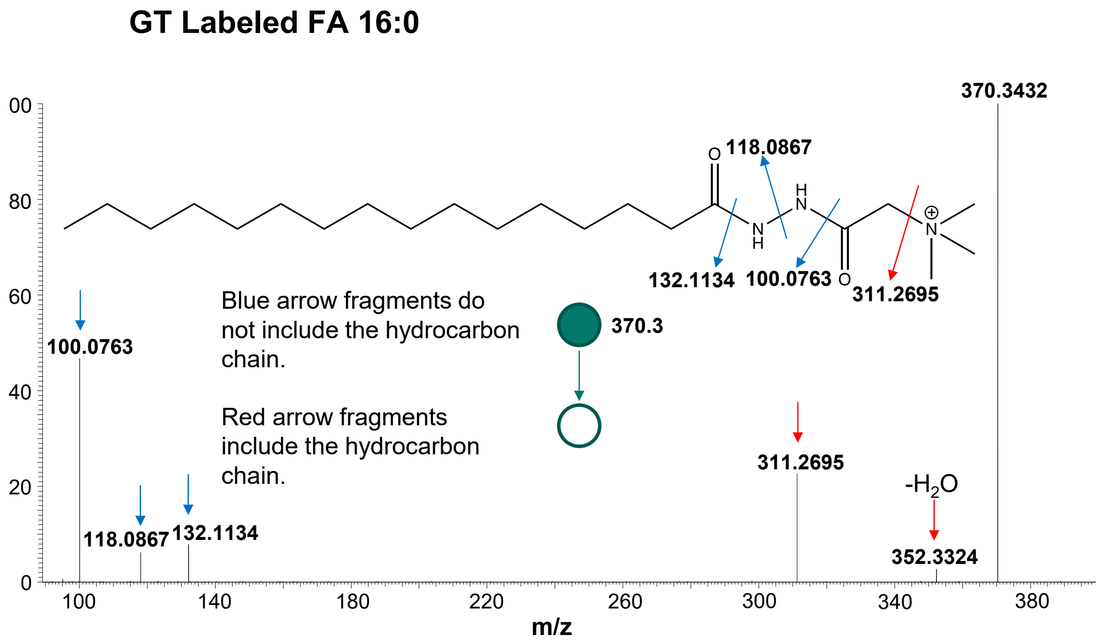

Figure S5. MS/MS spectrum of GT labeled FA 16:0 from tissue extract solution

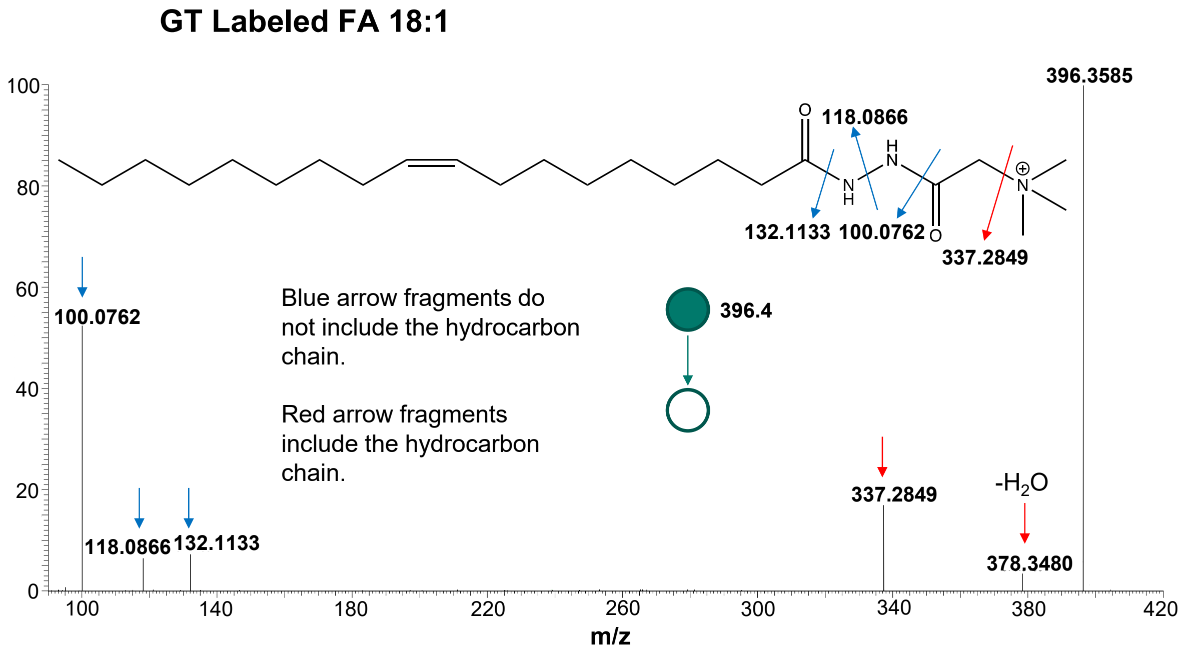

Figure S6. MS/MS spectrum of GT labeled FA 18:1 from tissue extract solution

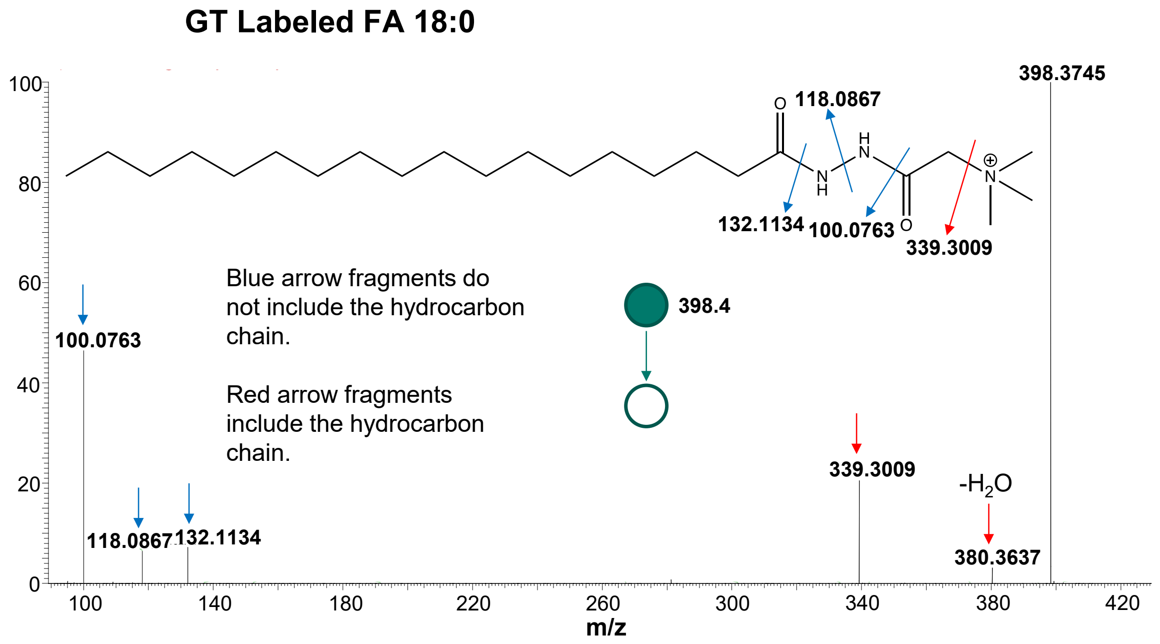

Figure S7. MS/MS spectrum of GT labeled FA 18:0 from tissue extract solution

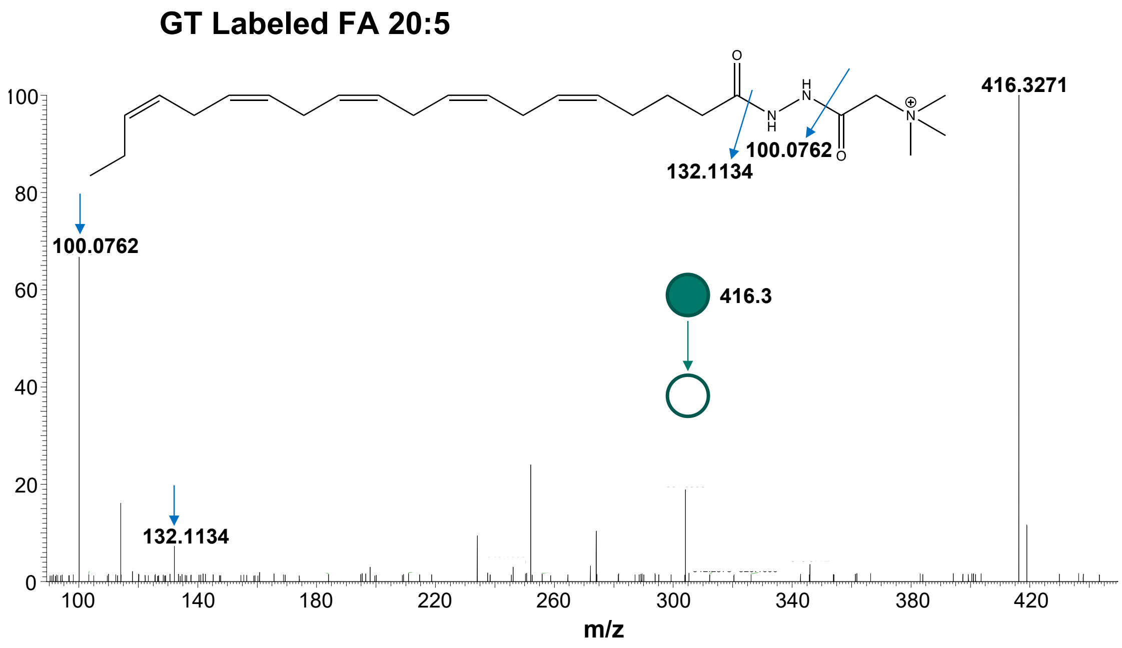

Figure S8. MS/MS spectrum of GT labeled FA 20:5 from tissue extract solution

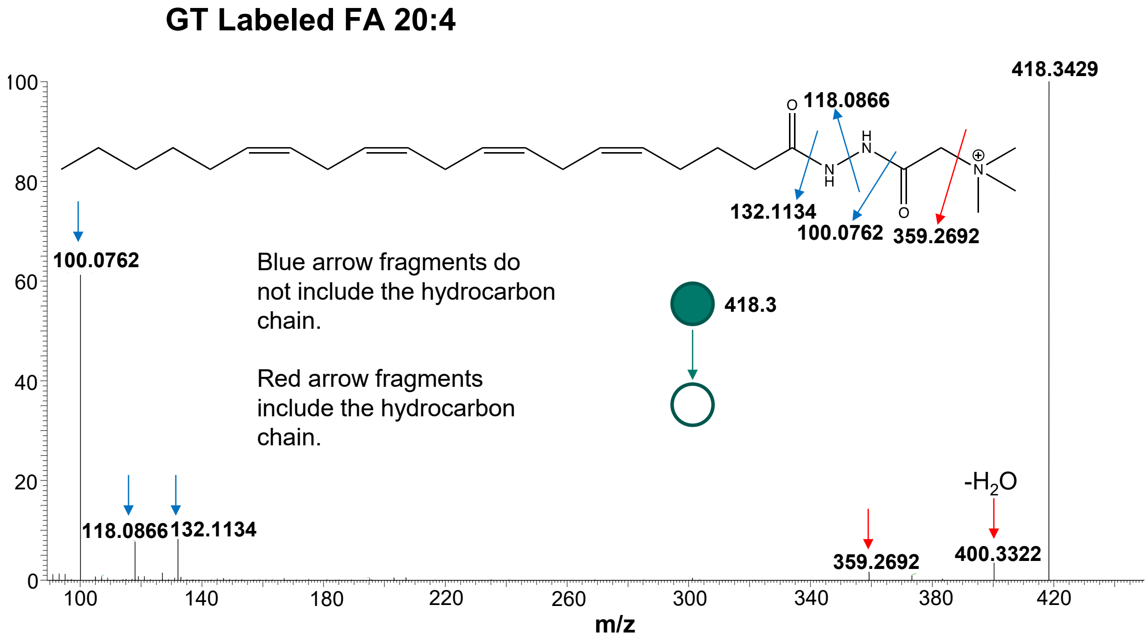

Figure S9. MS/MS spectrum of GT labeled FA 20:4 from tissue extract solution

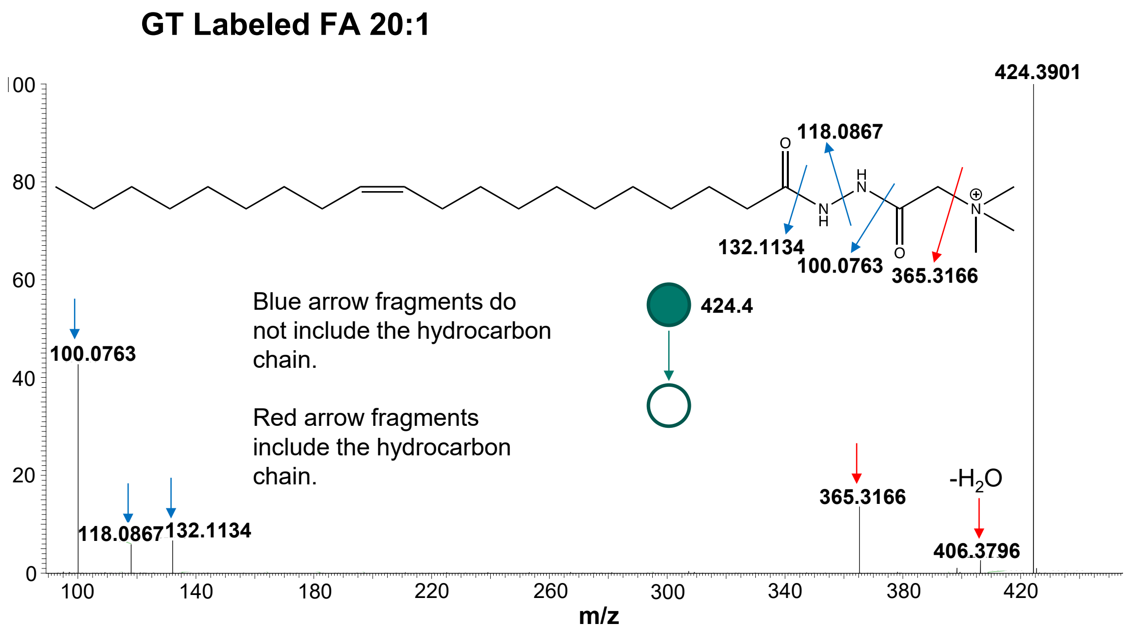

Figure S10. MS/MS spectrum of GT labeled FA 20:1 from tissue extract solution

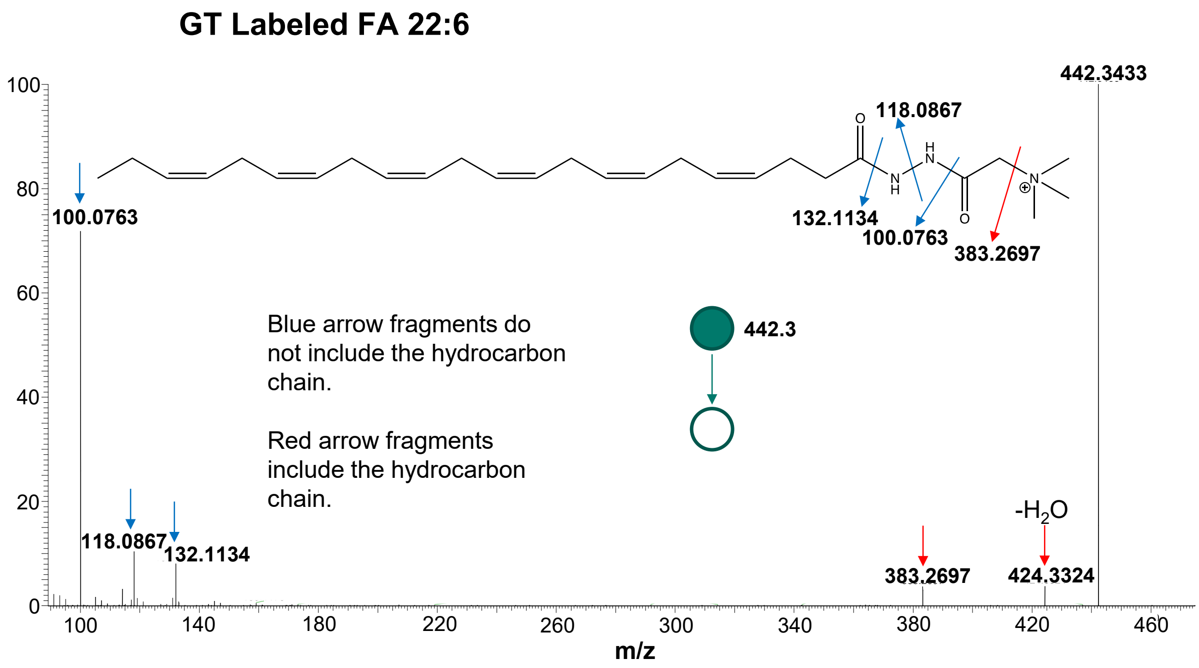

Figure S11. MS/MS spectrum of GT labeled FA 22:6 from tissue extract solution

Table S7. Retention times for different GT labeled FAs.

FAs	Precursor m/z	RT (min)
C16:1	368.3272	60.4
C16:0	370.3428	66.1
C18:1	396.3585	67.2
C18:0	398.3741	71.3
C20:5	416.3272	59.1
C20:4	418.3428	62.8
C20:1	424.3898	71.5
C22:6	442.3428	63.3

Table S8. Signal intensities and mass errors for different GT labeled FAs at extraction spot 1.

FAs	Signal Intensity	Mass Error (PPM)
C16:1	5.02E+07	1.8
C16:0	9.68E+08	1.5
C18:1	5.51E+08	1.7
C18:0	1.47E+10	1.1
C20:5	2.41E+05	1.7
C20:4	6.22E+08	1.2
C20:1	7.50E+09	1.4
C22:6	2.19E+07	2.3

Table S9. Signal intensities and mass errors for different GT labeled FAs at extraction spot 2.

FAs	Signal Intensity	Mass Error (PPM)
C16:1	9.90E+07	1.5
C16:0	2.10E+09	0.5
C18:1	2.83E+09	0.8
C18:0	1.60E+10	0.5
C20:5	9.13E+04	1.4
C20:4	1.15E+09	0.3
C20:1	1.73E+10	0.7
C22:6	4.70E+06	2.4

Table S10. Signal intensities and mass errors for different GT labeled FAs at extraction spot 3.

FAs	Signal Intensity	Mass Error (PPM)
C16:1	2.01E+07	1.7
C16:0	3.52E+08	1.1
C18:1	3.43E+08	1.1
C18:0	7.55E+09	0.4
C20:5	2.08E+05	0.8
C20:4	2.66E+08	0.8
C20:1	1.09E+10	0.6
C22:6	2.03E+07	1.4

Table S11. Signal intensities and mass errors for different GT labeled FAs at extraction spot 4.

FAs	Signal Intensity	Mass Error (PPM)
C16:1	5.82E+06	3.1
C16:0	9.46E+07	1.2
C18:1	6.59E+07	1.3
C18:0	1.56E+09	0
C20:5	1.99E+04	1.1
C20:4	5.70E+07	1.5
C20:1	3.51E+09	0
C22:6	7.15E+06	1.6
